# Pinnatane A from the bark of *Walsura pinnata* Hassk

**DOI:** 10.1107/S1600536809015955

**Published:** 2009-05-20

**Authors:** Khalit Mohamad, Mahfizah Yusoff, Khalijah Awang, Kartini Ahmad, Seik Weng Ng

**Affiliations:** a Department of Chemistry, University of Malaya, 50603 Kuala Lumpur, Malaysia

## Abstract

In the mol­ecule of pinnatane A, C_30_H_48_O_3_, isolated from the bark of *Walsura pinnata* Hassk, the four cyclo­hexane rings adopt chair conformations; the carboxyl and hydr­oxy substituents occupy axial positions. The cyclo­hexene ring is envelope-shaped. Adjacent mol­ecules are linked by O—H⋯O hydrogen bonds into a chain running along the *c* axis.

## Related literature

For related structures, see: Awang *et al.* (2009 ▶); Jiang *et al.* (1995 ▶).
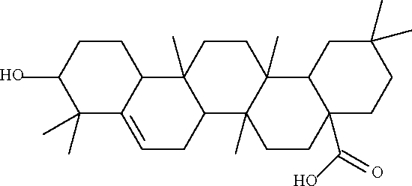


## Experimental

### 

#### Crystal data

C_30_H_48_O_3_
*M* *_r_* = 456.68
Orthorhombic, 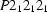

*a* = 7.3761 (2) Å
*b* = 16.3585 (4) Å
*c* = 20.7032 (5) Å
*V* = 2498.1 (1) Å^3^
*Z* = 4
Mo *K*α radiation
μ = 0.08 mm^−1^
*T* = 100 K
0.40 × 0.15 × 0.05 mm

#### Data collection

Bruker SMART APEX diffractometer
Absorption correction: none
17614 measured reflections
3268 independent reflections
2881 reflections with *I* > 2σ(*I*)
*R*_int_ = 0.045

#### Refinement

*R*[*F* ^2^ > 2σ(*F* ^2^)] = 0.037
*wR*(*F* ^2^) = 0.097
*S* = 1.00
3268 reflections
313 parameters
2 restraints
H atoms treated by a mixture of independent and constrained refinement
Δρ_max_ = 0.27 e Å^−3^
Δρ_min_ = −0.17 e Å^−3^

### 

Data collection: *APEX2* (Bruker, 2007 ▶); cell refinement: *SAINT* (Bruker, 2007 ▶); data reduction: *SAINT*; program(s) used to solve structure: *SHELXS97* (Sheldrick, 2008 ▶); program(s) used to refine structure: *SHELXL97* (Sheldrick, 2008 ▶); molecular graphics: *X-SEED* (Barbour, 2001 ▶); software used to prepare material for publication: *publCIF* (Westrip, 2009 ▶).

## Supplementary Material

Crystal structure: contains datablocks I, global. DOI: 10.1107/S1600536809015955/wn2325sup1.cif

Structure factors: contains datablocks I. DOI: 10.1107/S1600536809015955/wn2325Isup2.hkl

Additional supplementary materials:  crystallographic information; 3D view; checkCIF report

## Figures and Tables

**Table 1:** Hydrogen-bond geometry (Å, °)

*D*—H⋯*A*	*D*—H	H⋯*A*	*D*⋯*A*	*D*—H⋯*A*
O1—H1⋯O3^i^	0.85 (1)	1.90 (1)	2.731 (2)	167 (3)
O3—H3⋯O2^ii^	0.84 (1)	2.36 (2)	3.080 (2)	144 (2)

Symmetry codes: (i) 
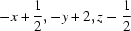
; (ii) 
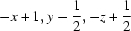
.

**Fig. 1.**
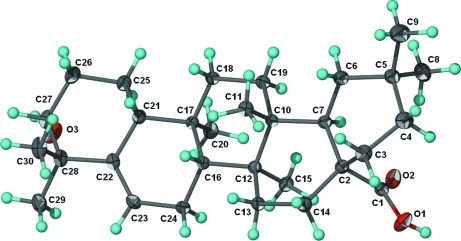
The molecular structure of the title compound. Displacement ellipsoids are drawn at the 70% probability level. Hydrogen atoms are drawn as spheres of arbitrary radius.

## supplementary crystallographic information

### Comment

Chemicals from *Walsura pinnata* Hassk have not hitherto been reported. We have recently reported the structure of 3-oxoolean-1-en-28-oic acid (Awang *et al.*, 2009), which was obtained from one fraction of the crude extract of the bark of this plant. The last fraction yielded the title compound, which we have named pinnatane A. A related carbon skeleton, assigned from spectroscopic measurements, has been reported (Jiang *et al.*, 1995).

In the molecule of pinnatane A (Fig. 1) the four cyclohexane rings adopt chair comformations, with axial carboxylic acid and hydroxy substituents. The cyclohexene ring is envelope-shaped. Adjacent molecules are linked by O—H···O hydrogen bonds into a chain running along the longest axis of the orthorhombic unit cell.

### Experimental

The dried and ground bark of *Walsura pinnata* Hassk (2.3 kg) was extracted with *n*-hexane for 72 h at room temperature. The solvent was evaporated to give a crude extract, which was subjected to column chromatography on silica gel (60 GF254), using *n*-hexane with increasing amounts of ethyl acetate as eluent. Of the twenty-four fractions collected, the twenty-fourth fraction, eluted with ethyl acetate:*n*-hexane (14:86) gave 2 g of the product, which was further purified by column chromatography (*n*-hexane:acetone, 94:6) to give the title compound (5 mg). The formulation was established by satisfactory solution NMR spectroscopy.

### Refinement

Carbon-bound H-atoms were placed in calculated positions (C—H 0.95–1.00 Å) and were included in the refinement in the riding model approximation, with *U*_iso_(H) set to 1.2–1.5*U*_eq_(C). The oxygen-bound H-atoms were located in a difference Fourier map, and were refined with a distance restraint of 0.84±0.01 Å; their displacement parameters were freely refined. In the absence of significant anomalous scattering effects, Friedel pairs were merged.

### Crystal data

**Table:**

C_30_H_48_O_3_	*F*(000) = 1008
*M**_r_* = 456.68	*D*_x_ = 1.214 Mg m^−^^3^
Orthorhombic, *P*2_1_2_1_2_1_	Mo *K*α radiation, λ = 0.71073 Å
Hall symbol: P 2ac 2ab	Cell parameters from 3887 reflections
*a* = 7.3761 (2) Å	θ = 2.3–28.0°
*b* = 16.3585 (4) Å	µ = 0.08 mm^−^^1^
*c* = 20.7032 (5) Å	*T* = 100 K
*V* = 2498.1 (1) Å^3^	Chip, colorless
*Z* = 4	0.40 × 0.15 × 0.05 mm

### Data collection

**Table:**

Bruker SMART APEX diffractometer	2881 reflections with *I* > 2σ(*I*)
Radiation source: fine-focus sealed tube	*R*_int_ = 0.045
graphite	θ_max_ = 27.5°, θ_min_ = 1.6°
ω scans	*h* = −9→9
17614 measured reflections	*k* = −21→21
3268 independent reflections	*l* = −26→25

### Refinement

**Table:**

Refinement on *F*^2^	Primary atom site location: structure-invariant direct methods
Least-squares matrix: full	Secondary atom site location: difference Fourier map
*R*[*F*^2^ > 2σ(*F*^2^)] = 0.037	Hydrogen site location: inferred from neighbouring sites
*wR*(*F*^2^) = 0.097	H atoms treated by a mixture of independent and constrained refinement
*S* = 1.00	*w* = 1/[σ^2^(*F*_o_^2^) + (0.0608*P*)^2^ + 0.3281*P*] where *P* = (*F*_o_^2^ + 2*F*_c_^2^)/3
3268 reflections	(Δ/σ)_max_ = 0.001
313 parameters	Δρ_max_ = 0.27 e Å^−^^3^
2 restraints	Δρ_min_ = −0.17 e Å^−^^3^

### Fractional atomic coordinates and isotropic or equivalent isotropic displacement parameters (Å^2^)

**Table:**

	*x*	*y*	*z*	*U*_iso_*/*U*_eq_	
O1	0.0192 (2)	1.08616 (9)	−0.04603 (8)	0.0226 (3)	
H1	0.022 (5)	1.1379 (6)	−0.0479 (15)	0.054 (9)*	
O2	0.2787 (2)	1.10407 (8)	0.00673 (6)	0.0191 (3)	
O3	0.5137 (2)	0.74943 (9)	0.43439 (7)	0.0219 (3)	
H3	0.590 (3)	0.7286 (16)	0.4595 (11)	0.040 (8)*	
C1	0.1662 (3)	1.05839 (11)	−0.01560 (9)	0.0145 (4)	
C2	0.1730 (3)	0.96355 (11)	−0.01453 (9)	0.0131 (4)	
C3	0.1421 (3)	0.93705 (12)	−0.08573 (9)	0.0168 (4)	
H3A	0.1447	0.8766	−0.0884	0.020*	
H3B	0.0208	0.9556	−0.0999	0.020*	
C4	0.2849 (3)	0.97201 (13)	−0.13099 (10)	0.0209 (4)	
H4A	0.2695	1.0321	−0.1333	0.025*	
H4B	0.2650	0.9497	−0.1749	0.025*	
C5	0.4827 (3)	0.95261 (13)	−0.10968 (9)	0.0197 (4)	
C6	0.4780 (3)	0.90007 (12)	−0.04821 (9)	0.0181 (4)	
H6A	0.6042	0.8920	−0.0332	0.022*	
H6B	0.4290	0.8456	−0.0596	0.022*	
C7	0.3656 (3)	0.93451 (11)	0.00866 (9)	0.0129 (4)	
H7	0.4306	0.9844	0.0242	0.015*	
C8	0.5911 (3)	1.03113 (14)	−0.09846 (11)	0.0267 (5)	
H8A	0.5294	1.0649	−0.0661	0.040*	
H8B	0.6005	1.0616	−0.1391	0.040*	
H8C	0.7129	1.0172	−0.0830	0.040*	
C9	0.5782 (4)	0.90337 (16)	−0.16263 (10)	0.0323 (6)	
H9A	0.5127	0.8521	−0.1699	0.049*	
H9B	0.7028	0.8913	−0.1492	0.049*	
H9C	0.5802	0.9352	−0.2027	0.049*	
C10	0.3632 (3)	0.87291 (11)	0.06662 (9)	0.0118 (4)	
C11	0.3079 (3)	0.78698 (11)	0.04270 (9)	0.0159 (4)	
H11A	0.4104	0.7616	0.0203	0.024*	
H11B	0.2051	0.7918	0.0129	0.024*	
H11C	0.2729	0.7531	0.0797	0.024*	
C12	0.2315 (3)	0.90314 (11)	0.12067 (8)	0.0116 (4)	
C13	0.0361 (3)	0.89617 (12)	0.09446 (9)	0.0148 (4)	
H13A	−0.0464	0.9250	0.1245	0.018*	
H13B	0.0009	0.8378	0.0943	0.018*	
C14	0.0073 (3)	0.93116 (12)	0.02569 (9)	0.0172 (4)	
H14A	−0.0805	0.9767	0.0294	0.021*	
H14B	−0.0520	0.8880	−0.0003	0.021*	
C15	0.2605 (3)	0.99440 (11)	0.13609 (9)	0.0151 (4)	
H15A	0.2138	1.0063	0.1794	0.023*	
H15B	0.1958	1.0278	0.1043	0.023*	
H15C	0.3902	1.0071	0.1344	0.023*	
C16	0.2508 (3)	0.84795 (11)	0.18263 (9)	0.0116 (4)	
H16	0.2122	0.7920	0.1688	0.014*	
C17	0.4463 (3)	0.83731 (11)	0.20946 (9)	0.0126 (4)	
C18	0.5718 (3)	0.81296 (12)	0.15331 (9)	0.0147 (4)	
H18A	0.6988	0.8135	0.1688	0.018*	
H18B	0.5427	0.7563	0.1400	0.018*	
C19	0.5566 (3)	0.86876 (12)	0.09454 (9)	0.0147 (4)	
H19A	0.6402	0.8489	0.0606	0.018*	
H19B	0.5955	0.9245	0.1069	0.018*	
C20	0.5197 (3)	0.91447 (11)	0.24330 (10)	0.0168 (4)	
H20A	0.4518	0.9239	0.2833	0.025*	
H20B	0.5057	0.9617	0.2146	0.025*	
H20C	0.6484	0.9068	0.2535	0.025*	
C21	0.4437 (3)	0.76507 (11)	0.25864 (9)	0.0136 (4)	
H21	0.4314	0.7139	0.2325	0.016*	
C22	0.2839 (3)	0.76640 (11)	0.30481 (9)	0.0139 (4)	
C23	0.1435 (3)	0.81574 (12)	0.29524 (9)	0.0150 (4)	
H23	0.0502	0.8161	0.3269	0.018*	
C24	0.1219 (3)	0.87123 (12)	0.23765 (9)	0.0151 (4)	
H24A	0.1461	0.9283	0.2509	0.018*	
H24B	−0.0047	0.8682	0.2220	0.018*	
C25	0.6225 (3)	0.75728 (12)	0.29622 (10)	0.0182 (4)	
H25A	0.7252	0.7543	0.2656	0.022*	
H25B	0.6397	0.8060	0.3239	0.022*	
C26	0.6192 (3)	0.68067 (12)	0.33802 (10)	0.0191 (4)	
H26A	0.5993	0.6321	0.3104	0.023*	
H26B	0.7377	0.6742	0.3599	0.023*	
C27	0.4700 (3)	0.68582 (11)	0.38820 (9)	0.0168 (4)	
H27	0.4658	0.6325	0.4118	0.020*	
C28	0.2819 (3)	0.70122 (11)	0.35883 (9)	0.0156 (4)	
C29	0.1487 (3)	0.72131 (13)	0.41332 (9)	0.0199 (4)	
H29A	0.1724	0.7766	0.4294	0.030*	
H29B	0.1641	0.6820	0.4486	0.030*	
H29C	0.0243	0.7182	0.3969	0.030*	
C30	0.2190 (3)	0.62008 (12)	0.32797 (10)	0.0215 (4)	
H30A	0.3000	0.6059	0.2922	0.032*	
H30B	0.0950	0.6263	0.3117	0.032*	
H30C	0.2221	0.5766	0.3605	0.032*	

### Atomic displacement parameters (Å^2^)

**Table:**

	*U*^11^	*U*^22^	*U*^33^	*U*^12^	*U*^13^	*U*^23^
O1	0.0198 (8)	0.0148 (7)	0.0332 (8)	0.0020 (6)	−0.0087 (7)	0.0031 (6)
O2	0.0224 (8)	0.0151 (6)	0.0198 (7)	−0.0031 (6)	−0.0050 (6)	0.0014 (5)
O3	0.0293 (9)	0.0174 (7)	0.0190 (7)	0.0024 (7)	−0.0108 (7)	−0.0029 (6)
C1	0.0152 (10)	0.0161 (9)	0.0121 (8)	0.0021 (8)	0.0016 (8)	0.0006 (7)
C2	0.0123 (9)	0.0140 (8)	0.0130 (9)	0.0006 (7)	−0.0012 (8)	0.0005 (7)
C3	0.0186 (10)	0.0184 (9)	0.0134 (9)	0.0001 (8)	−0.0033 (8)	−0.0014 (7)
C4	0.0216 (11)	0.0284 (11)	0.0126 (9)	0.0043 (9)	−0.0013 (9)	0.0004 (8)
C5	0.0208 (11)	0.0254 (10)	0.0128 (9)	0.0072 (9)	0.0044 (9)	0.0035 (8)
C6	0.0184 (10)	0.0223 (10)	0.0136 (9)	0.0065 (9)	0.0025 (8)	0.0030 (8)
C7	0.0119 (9)	0.0146 (9)	0.0121 (8)	0.0012 (7)	0.0002 (7)	0.0005 (7)
C8	0.0181 (11)	0.0348 (12)	0.0273 (11)	−0.0002 (10)	0.0018 (9)	0.0123 (10)
C9	0.0379 (15)	0.0426 (14)	0.0165 (10)	0.0175 (12)	0.0082 (10)	0.0050 (10)
C10	0.0106 (9)	0.0128 (8)	0.0119 (8)	0.0012 (7)	0.0012 (7)	0.0009 (7)
C11	0.0175 (10)	0.0141 (9)	0.0160 (9)	0.0010 (8)	0.0010 (8)	−0.0008 (7)
C12	0.0111 (9)	0.0126 (8)	0.0112 (8)	0.0006 (7)	−0.0005 (7)	0.0001 (6)
C13	0.0120 (9)	0.0170 (9)	0.0155 (9)	0.0012 (8)	0.0008 (8)	0.0034 (7)
C14	0.0131 (10)	0.0195 (9)	0.0190 (9)	−0.0007 (8)	−0.0017 (8)	0.0045 (8)
C15	0.0166 (10)	0.0142 (9)	0.0144 (8)	0.0014 (8)	−0.0006 (8)	0.0004 (7)
C16	0.0114 (9)	0.0105 (8)	0.0129 (8)	−0.0003 (7)	0.0009 (7)	0.0016 (6)
C17	0.0115 (9)	0.0135 (9)	0.0128 (9)	0.0009 (7)	0.0001 (7)	0.0018 (7)
C18	0.0116 (10)	0.0172 (9)	0.0151 (9)	0.0023 (8)	0.0009 (7)	0.0031 (7)
C19	0.0128 (10)	0.0167 (9)	0.0146 (9)	0.0005 (8)	0.0008 (8)	0.0014 (7)
C20	0.0169 (10)	0.0160 (9)	0.0175 (9)	−0.0023 (8)	−0.0021 (8)	0.0013 (7)
C21	0.0147 (10)	0.0128 (9)	0.0134 (9)	0.0008 (7)	−0.0001 (7)	0.0007 (7)
C22	0.0168 (10)	0.0136 (8)	0.0114 (8)	−0.0035 (7)	−0.0014 (8)	−0.0003 (7)
C23	0.0141 (10)	0.0187 (9)	0.0122 (8)	−0.0008 (8)	0.0021 (8)	−0.0002 (7)
C24	0.0121 (10)	0.0184 (9)	0.0150 (9)	0.0025 (8)	0.0015 (8)	0.0025 (7)
C25	0.0152 (10)	0.0188 (10)	0.0205 (10)	0.0013 (8)	−0.0003 (8)	0.0053 (8)
C26	0.0206 (11)	0.0184 (10)	0.0183 (9)	0.0033 (9)	−0.0030 (9)	0.0032 (8)
C27	0.0245 (11)	0.0126 (9)	0.0133 (9)	0.0000 (8)	−0.0028 (8)	0.0001 (7)
C28	0.0198 (10)	0.0137 (9)	0.0131 (9)	−0.0019 (8)	−0.0007 (8)	−0.0002 (7)
C29	0.0225 (11)	0.0236 (10)	0.0136 (9)	−0.0015 (9)	0.0011 (8)	0.0038 (8)
C30	0.0281 (12)	0.0192 (10)	0.0172 (9)	−0.0063 (9)	−0.0039 (9)	0.0020 (8)

### Geometric parameters (Å, °)

**Table:**

O1—C1	1.334 (2)	C15—H15A	0.9800
O1—H1	0.85 (1)	C15—H15B	0.9800
O2—C1	1.208 (2)	C15—H15C	0.9800
O3—C27	1.450 (2)	C16—C24	1.532 (3)
O3—H3	0.84 (1)	C16—C17	1.555 (3)
C1—C2	1.552 (3)	C16—H16	1.0000
C2—C3	1.553 (3)	C17—C18	1.538 (3)
C2—C14	1.571 (3)	C17—C20	1.542 (3)
C2—C7	1.573 (3)	C17—C21	1.560 (2)
C3—C4	1.521 (3)	C18—C19	1.525 (2)
C3—H3A	0.9900	C18—H18A	0.9900
C3—H3B	0.9900	C18—H18B	0.9900
C4—C5	1.557 (3)	C19—H19A	0.9900
C4—H4A	0.9900	C19—H19B	0.9900
C4—H4B	0.9900	C20—H20A	0.9800
C5—C9	1.532 (3)	C20—H20B	0.9800
C5—C8	1.531 (3)	C20—H20C	0.9800
C5—C6	1.536 (3)	C21—C22	1.518 (3)
C6—C7	1.546 (3)	C21—C25	1.536 (3)
C6—H6A	0.9900	C21—H21	1.0000
C6—H6B	0.9900	C22—C23	1.328 (3)
C7—C10	1.567 (2)	C22—C28	1.545 (2)
C7—H7	1.0000	C23—C24	1.507 (2)
C8—H8A	0.9800	C23—H23	0.9500
C8—H8B	0.9800	C24—H24A	0.9900
C8—H8C	0.9800	C24—H24B	0.9900
C9—H9A	0.9800	C25—C26	1.523 (3)
C9—H9B	0.9800	C25—H25A	0.9900
C9—H9C	0.9800	C25—H25B	0.9900
C10—C19	1.541 (3)	C26—C27	1.516 (3)
C10—C11	1.545 (3)	C26—H26A	0.9900
C10—C12	1.562 (3)	C26—H26B	0.9900
C11—H11A	0.9800	C27—C28	1.535 (3)
C11—H11B	0.9800	C27—H27	1.0000
C11—H11C	0.9800	C28—C29	1.532 (3)
C12—C15	1.542 (3)	C28—C30	1.544 (3)
C12—C13	1.544 (3)	C29—H29A	0.9800
C12—C16	1.575 (2)	C29—H29B	0.9800
C13—C14	1.549 (3)	C29—H29C	0.9800
C13—H13A	0.9900	C30—H30A	0.9800
C13—H13B	0.9900	C30—H30B	0.9800
C14—H14A	0.9900	C30—H30C	0.9800
C14—H14B	0.9900		
			
C1—O1—H1	110 (2)	H15A—C15—H15C	109.5
C27—O3—H3	105.5 (19)	H15B—C15—H15C	109.5
O2—C1—O1	121.87 (17)	C24—C16—C17	109.75 (15)
O2—C1—C2	126.19 (18)	C24—C16—C12	114.01 (15)
O1—C1—C2	111.93 (17)	C17—C16—C12	116.04 (15)
C1—C2—C3	105.10 (15)	C24—C16—H16	105.3
C1—C2—C14	108.63 (16)	C17—C16—H16	105.3
C3—C2—C14	107.14 (16)	C12—C16—H16	105.3
C1—C2—C7	109.60 (16)	C18—C17—C20	110.12 (16)
C3—C2—C7	109.74 (16)	C18—C17—C16	108.48 (15)
C14—C2—C7	116.04 (15)	C20—C17—C16	113.35 (16)
C4—C3—C2	112.22 (16)	C18—C17—C21	107.73 (15)
C4—C3—H3A	109.2	C20—C17—C21	109.15 (15)
C2—C3—H3A	109.2	C16—C17—C21	107.84 (15)
C4—C3—H3B	109.2	C19—C18—C17	113.81 (15)
C2—C3—H3B	109.2	C19—C18—H18A	108.8
H3A—C3—H3B	107.9	C17—C18—H18A	108.8
C3—C4—C5	113.42 (16)	C19—C18—H18B	108.8
C3—C4—H4A	108.9	C17—C18—H18B	108.8
C5—C4—H4A	108.9	H18A—C18—H18B	107.7
C3—C4—H4B	108.9	C18—C19—C10	113.18 (16)
C5—C4—H4B	108.9	C18—C19—H19A	108.9
H4A—C4—H4B	107.7	C10—C19—H19A	108.9
C9—C5—C8	108.03 (19)	C18—C19—H19B	108.9
C9—C5—C6	108.00 (16)	C10—C19—H19B	108.9
C8—C5—C6	110.83 (17)	H19A—C19—H19B	107.8
C9—C5—C4	109.59 (18)	C17—C20—H20A	109.5
C8—C5—C4	111.19 (17)	C17—C20—H20B	109.5
C6—C5—C4	109.13 (17)	H20A—C20—H20B	109.5
C5—C6—C7	116.03 (16)	C17—C20—H20C	109.5
C5—C6—H6A	108.3	H20A—C20—H20C	109.5
C7—C6—H6A	108.3	H20B—C20—H20C	109.5
C5—C6—H6B	108.3	C22—C21—C25	110.42 (15)
C7—C6—H6B	108.3	C22—C21—C17	114.20 (15)
H6A—C6—H6B	107.4	C25—C21—C17	112.50 (16)
C6—C7—C10	110.77 (15)	C22—C21—H21	106.4
C6—C7—C2	111.23 (15)	C25—C21—H21	106.4
C10—C7—C2	114.71 (15)	C17—C21—H21	106.4
C6—C7—H7	106.5	C23—C22—C21	121.36 (16)
C10—C7—H7	106.5	C23—C22—C28	121.35 (18)
C2—C7—H7	106.5	C21—C22—C28	116.93 (16)
C5—C8—H8A	109.5	C22—C23—C24	124.52 (18)
C5—C8—H8B	109.5	C22—C23—H23	117.7
H8A—C8—H8B	109.5	C24—C23—H23	117.7
C5—C8—H8C	109.5	C23—C24—C16	111.90 (16)
H8A—C8—H8C	109.5	C23—C24—H24A	109.2
H8B—C8—H8C	109.5	C16—C24—H24A	109.2
C5—C9—H9A	109.5	C23—C24—H24B	109.2
C5—C9—H9B	109.5	C16—C24—H24B	109.2
H9A—C9—H9B	109.5	H24A—C24—H24B	107.9
C5—C9—H9C	109.5	C26—C25—C21	110.02 (17)
H9A—C9—H9C	109.5	C26—C25—H25A	109.7
H9B—C9—H9C	109.5	C21—C25—H25A	109.7
C19—C10—C11	108.97 (16)	C26—C25—H25B	109.7
C19—C10—C12	108.72 (14)	C21—C25—H25B	109.7
C11—C10—C12	110.67 (16)	H25A—C25—H25B	108.2
C19—C10—C7	107.78 (15)	C27—C26—C25	110.80 (17)
C11—C10—C7	110.03 (15)	C27—C26—H26A	109.5
C12—C10—C7	110.60 (14)	C25—C26—H26A	109.5
C10—C11—H11A	109.5	C27—C26—H26B	109.5
C10—C11—H11B	109.5	C25—C26—H26B	109.5
H11A—C11—H11B	109.5	H26A—C26—H26B	108.1
C10—C11—H11C	109.5	O3—C27—C26	109.29 (17)
H11A—C11—H11C	109.5	O3—C27—C28	110.14 (16)
H11B—C11—H11C	109.5	C26—C27—C28	113.18 (16)
C15—C12—C13	105.88 (16)	O3—C27—H27	108.0
C15—C12—C10	111.64 (15)	C26—C27—H27	108.0
C13—C12—C10	107.79 (14)	C28—C27—H27	108.0
C15—C12—C16	111.95 (14)	C29—C28—C27	108.85 (15)
C13—C12—C16	109.17 (15)	C29—C28—C30	107.23 (17)
C10—C12—C16	110.22 (14)	C27—C28—C30	107.11 (16)
C12—C13—C14	115.06 (16)	C29—C28—C22	113.01 (16)
C12—C13—H13A	108.5	C27—C28—C22	113.06 (16)
C14—C13—H13A	108.5	C30—C28—C22	107.25 (15)
C12—C13—H13B	108.5	C28—C29—H29A	109.5
C14—C13—H13B	108.5	C28—C29—H29B	109.5
H13A—C13—H13B	107.5	H29A—C29—H29B	109.5
C13—C14—C2	120.34 (17)	C28—C29—H29C	109.5
C13—C14—H14A	107.2	H29A—C29—H29C	109.5
C2—C14—H14A	107.2	H29B—C29—H29C	109.5
C13—C14—H14B	107.2	C28—C30—H30A	109.5
C2—C14—H14B	107.2	C28—C30—H30B	109.5
H14A—C14—H14B	106.9	H30A—C30—H30B	109.5
C12—C15—H15A	109.5	C28—C30—H30C	109.5
C12—C15—H15B	109.5	H30A—C30—H30C	109.5
H15A—C15—H15B	109.5	H30B—C30—H30C	109.5
C12—C15—H15C	109.5		
			
O2—C1—C2—C3	−128.3 (2)	C15—C12—C16—C17	−72.0 (2)
O1—C1—C2—C3	50.5 (2)	C13—C12—C16—C17	171.15 (15)
O2—C1—C2—C14	117.3 (2)	C10—C12—C16—C17	52.9 (2)
O1—C1—C2—C14	−63.9 (2)	C24—C16—C17—C18	179.31 (15)
O2—C1—C2—C7	−10.4 (3)	C12—C16—C17—C18	−49.6 (2)
O1—C1—C2—C7	168.37 (15)	C24—C16—C17—C20	−58.05 (19)
C1—C2—C3—C4	59.0 (2)	C12—C16—C17—C20	72.99 (19)
C14—C2—C3—C4	174.41 (16)	C24—C16—C17—C21	62.89 (19)
C7—C2—C3—C4	−58.8 (2)	C12—C16—C17—C21	−166.06 (14)
C2—C3—C4—C5	54.0 (2)	C20—C17—C18—C19	−73.7 (2)
C3—C4—C5—C9	119.69 (19)	C16—C17—C18—C19	50.8 (2)
C3—C4—C5—C8	−120.95 (19)	C21—C17—C18—C19	167.33 (16)
C3—C4—C5—C6	1.6 (2)	C17—C18—C19—C10	−58.2 (2)
C9—C5—C6—C7	−172.55 (19)	C11—C10—C19—C18	−62.8 (2)
C8—C5—C6—C7	69.3 (2)	C12—C10—C19—C18	57.9 (2)
C4—C5—C6—C7	−53.5 (2)	C7—C10—C19—C18	177.82 (15)
C5—C6—C7—C10	176.81 (17)	C18—C17—C21—C22	−161.11 (16)
C5—C6—C7—C2	48.0 (2)	C20—C17—C21—C22	79.3 (2)
C1—C2—C7—C6	−106.48 (18)	C16—C17—C21—C22	−44.2 (2)
C3—C2—C7—C6	8.5 (2)	C18—C17—C21—C25	72.02 (19)
C14—C2—C7—C6	130.06 (17)	C20—C17—C21—C25	−47.5 (2)
C1—C2—C7—C10	126.83 (16)	C16—C17—C21—C25	−171.08 (15)
C3—C2—C7—C10	−118.23 (16)	C25—C21—C22—C23	140.08 (18)
C14—C2—C7—C10	3.4 (2)	C17—C21—C22—C23	12.1 (2)
C6—C7—C10—C19	67.88 (19)	C25—C21—C22—C28	−46.6 (2)
C2—C7—C10—C19	−165.19 (16)	C17—C21—C22—C28	−174.56 (16)
C6—C7—C10—C11	−50.8 (2)	C21—C22—C23—C24	3.2 (3)
C2—C7—C10—C11	76.1 (2)	C28—C22—C23—C24	−169.77 (18)
C6—C7—C10—C12	−173.40 (16)	C22—C23—C24—C16	15.8 (3)
C2—C7—C10—C12	−46.5 (2)	C17—C16—C24—C23	−48.9 (2)
C19—C10—C12—C15	71.33 (18)	C12—C16—C24—C23	178.98 (15)
C11—C10—C12—C15	−169.02 (15)	C22—C21—C25—C26	57.1 (2)
C7—C10—C12—C15	−46.8 (2)	C17—C21—C25—C26	−173.99 (16)
C19—C10—C12—C13	−172.80 (15)	C21—C25—C26—C27	−62.7 (2)
C11—C10—C12—C13	−53.15 (19)	C25—C26—C27—O3	−67.6 (2)
C7—C10—C12—C13	69.05 (18)	C25—C26—C27—C28	55.6 (2)
C19—C10—C12—C16	−53.74 (19)	O3—C27—C28—C29	−46.8 (2)
C11—C10—C12—C16	65.91 (19)	C26—C27—C28—C29	−169.46 (16)
C7—C10—C12—C16	−171.90 (15)	O3—C27—C28—C30	−162.41 (16)
C15—C12—C13—C14	72.33 (19)	C26—C27—C28—C30	74.91 (19)
C10—C12—C13—C14	−47.3 (2)	O3—C27—C28—C22	79.67 (19)
C16—C12—C13—C14	−166.99 (15)	C26—C27—C28—C22	−43.0 (2)
C12—C13—C14—C2	5.1 (3)	C23—C22—C28—C29	−22.9 (3)
C1—C2—C14—C13	−106.01 (19)	C21—C22—C28—C29	163.82 (16)
C3—C2—C14—C13	140.93 (18)	C23—C22—C28—C27	−147.09 (18)
C7—C2—C14—C13	18.0 (2)	C21—C22—C28—C27	39.6 (2)
C15—C12—C16—C24	57.0 (2)	C23—C22—C28—C30	95.1 (2)
C13—C12—C16—C24	−59.8 (2)	C21—C22—C28—C30	−78.2 (2)
C10—C12—C16—C24	−178.05 (15)		

### Hydrogen-bond geometry (Å, °)

**Table:**

*D*—H···*A*	*D*—H	H···*A*	*D*···*A*	*D*—H···*A*
O1—H1···O3^i^	0.85 (1)	1.90 (1)	2.731 (2)	167 (3)
O3—H3···O2^ii^	0.84 (1)	2.36 (2)	3.080 (2)	144 (2)

Symmetry codes: (i) −*x*+1/2, −*y*+2, *z*−1/2; (ii) −*x*+1, *y*−1/2, −*z*+1/2.

## References

[bb1] Awang, K., Yusoff, M., Mohamad, K., Chong, S. L. & Ng, S. W. (2009). *Acta Cryst.* E**65**, o1166. 10.1107/S1600536809015086 PMC2977831 21583968

[bb2] Barbour, L. J. (2001). *J. Supramol. Chem.* **1**, 189–191.

[bb3] Bruker (2007). *APEX2* and *SAINT* Bruker AXS Inc., Madison, Wisconsin, USA.

[bb4] Jiang, Z.-H., Zhou, R.-H., Sasuda, K. & Ageta, H. (1995). *Phytochemistry*, **40**, 219–224.

[bb5] Sheldrick, G. M. (2008). *Acta Cryst.* A**64**, 112–122. 10.1107/S0108767307043930 18156677

[bb6] Westrip, S. P. (2009). *publCIF* In preparation.

